# Supplementary material for: Ecological and Construct Validity of a New Technical Level Cuban Dance Field Test
Source: Int J Environ Res Public Health. 2021 Dec 16;18(24):13287. doi: 10.3390/ijerph182413287 (PMC8701756; doi:10.3390/ijerph182413287)
Supplement: Supplementary file 1 [file ijerph-18-13287-s001.zip › ijerph-1466702-supplementary.pdf]

International Journal of
Environmental Research and Public Health

MDPI

Supplementary Materials

Table S1. ROC curves: Sensitivity (95% CI), specificity (95% CI), positive predictive value (PPV), negative predictive value (NPV), and accuracy on the basis of the level of difficulty (beginner vs. intermediate).

Threshold	Sensitivity	95% CI Sensitivity	Specificity	95% CI Specificity	PPV	NPV	Accuracy
- Inf.	1.000	1.000–1.000	0.000	0.000–0.000	0.500	-	0.500
27.5	1.000	1.000–1.000	0.033	0.000–0.100	0.508	1.000	0.517
29	1.000	1.000–1.000	0.100	0.000–0.233	0.526	1.000	0.550
33	1.000	1.000–1.000	0.133	0.033–0.267	0.536	1.000	0.567
37	1.000	1.000–1.000	0.167	0.033–0.300	0.545	1.000	0.583
39	1.000	1.000–1.000	0.300	0.133–0.467	0.588	1.000	0.650
49.5	1.000	1.000–1.000	0.333	0.167–0.500	0.600	1.000	0.667
77	1.000	1.000–1.000	0.367	0.200–0.533	0.612	1.000	0.683
96	1.000	1.000–1.000	0.400	0.233–0.567	0.625	1.000	0.700
98.5	1.000	1.000–1.000	0.467	0.300–0.634	0.652	1.000	0.733
100.5	1.000	1.000–1.000	0.633	0.467–0.800	0.732	1.000	0.817
140.5*	1.000	1.000–1.000	0.667	0.500–0.833	0.750	1.000	0.833
189	0.967	0.900–1.000	0.667	0.500–0.833	0.744	0.952	0.817
199	0.933	0.833–1.000	0.667	0.500–0.833	0.737	0.909	0.800
200.5	0.800	0.666–0.933	0.667	0.500–0.833	0.706	0.769	0.733
201.5	0.733	0.567–0.867	0.667	0.500–0.833	0.688	0.714	0.700
202.5	0.700	0.533–0.867	0.667	0.500–0.833	0.677	0.690	0.683
250	0.667	0.500–0.833	0.667	0.500–0.833	0.667	0.667	0.667
297.5	0.667	0.500–0.833	0.700	0.533–0.833	0.690	0.677	0.683
299	0.667	0.500–0.833	0.733	0.567–0.867	0.714	0.688	0.700
300.5	0.533	0.333–0.700	0.933	0.833–1.000	0.889	0.667	0.733
301.5	0.467	0.267–0.633	1.000	1.000–1.000	1.000	0.652	0.733
302.5	0.300	0.133–0.467	1.000	1.000–1.000	1.000	0.588	0.650
303.5	0.200	0.067–0.367	1.000	1.000–1.000	1.000	0.556	0.600
304.5	0.167	0.033–0.333	1.000	1.000–1.000	1.000	0.545	0.583
305.5	0.100	0.00–0.233	1.000	1.000–1.000	1.000	0.526	0.550
307	0.033	0.000–0.100	1.000	1.000–1.000	1.000	0.508	0.517
Inf.	0.000	0.000–0.000	1.000	1.000–1.000	-	0.500	0.500

* Highest average of sensitivity and specificity. *AUC* = 0.864; 95% CI = 0.864–0.954.

Table S2. ROC curves: Sensitivity (95% CI), specificity (95% CI), positive predictive value (PPV), negative predictive value (NPV), and accuracy on the basis of the level of difficulty (intermediate vs. advanced).

Threshold	Sensitivity	95% CI Sensitivity	Specificity	95% CI Specificity	PPV	NPV	Accuracy
-Inf	1.000	1.000–1.000	0.000	0.000–0.000	0.500	-	0.500
189	1.000	1.000–1.000	0.033	0.000–0.100	0.508	1.000	0.517
199	1.000	1.000–1.000	0.067	0.000–0.167	0.517	1.000	0.533
200.5	1.000	1.000–1.000	0.200	0.067–0.333	0.556	1.000	0.600
201.5	1.000	1.000–1.000	0.267	0.133–0.433	0.577	1.000	0.633
202.5	1.000	1.000–1.000	0.300	0.133–0.467	0.588	1.000	0.650
251.5	1.000	1.000–1.000	0.333	0.167–0.500	0.600	1.000	0.667
300.5	1.000	1.000–1.000	0.467	0.300–0.667	0.652	1.000	0.733
301.5	0.933	0.833–1.000	0.533	0.367–0.733	0.667	0.889	0.733
302.5	0.700	0.533–0.867	0.700	0.533–0.867	0.700	0.700	0.700
303.5	0.700	0.533–0.867	0.800	0.667–0.933	0.778	0.727	0.750
304.5	0.700	0.533–0.867	0.833	0.700–0.967	0.808	0.735	0.767
305.5*	0.700	0.533–0.867	0.900	0.800–1.000	0.875	0.750	0.800
306.5	0.633	0.467–0.800	0.967	0.900–1.000	0.950	0.725	0.800
307.5	0.367	0.200–0.533	0.967	0.900–1.000	0.917	0.604	0.667
308.5	0.367	0.200–0.533	1.000	1.000–1.000	1.000	0.612	0.683
314.5	0.333	0.167–0.500	1.000	1.000–1.000	1.000	0.600	0.667
Inf	0.000	0.000–0.000	1.000	1.000–1.000	-	0.500	0.500

* Highest average of sensitivity and specificity. *AUC* = 0.864; 95% CI = 0.864–0.952.

Table S3. ROC curve: Sensitivity (95% CI), specificity (95% CI), positive predictive value (PPV), negative predictive value (NPV), and accuracy on the basis of the level of difficulty (beginner vs. advanced).

Threshold	Sensitivity	95% CI Sensitivity	Specificity	95% CI Specificity	PPV	NPV	Accuracy
-Inf	1.000	1.000–1.000	0.000	0.000–0.000	0.500	-	0.500
27.5	1.000	1.000–1.000	0.033	0.000–0.100	0.508	1.000	0.517
29	1.000	1.000–1.000	0.100	0.000–0.233	0.526	1.000	0.550
33	1.000	1.000–1.000	0.133	0.033–0.267	0.536	1.000	0.567
37	1.000	1.000–1.000	0.167	0.033–0.300	0.545	1.000	0.583
39	1.000	1.000–1.000	0.300	0.133–0.467	0.588	1.000	0.650
49.5	1.000	1.000–1.000	0.333	0.167–0.500	0.600	1.000	0.667
77	1.000	1.000–1.000	0.367	0.200–0.533	0.612	1.000	0.683
96	1.000	1.000–1.000	0.400	0.233–0.567	0.625	1.000	0.700
98.5	1.000	1.000–1.000	0.467	0.300–0.633	0.652	1.000	0.733
100.5	1.000	1.000–1.000	0.633	0.433–0.800	0.732	1.000	0.817
199	1.000	1.000–1.000	0.667	0.500–0.833	0.750	1.000	0.833
297.5	1.000	1.000–1.000	0.700	0.533–0.867	0.769	1.000	0.850
299	1.000	1.000–1.000	0.733	0.567–0.900	0.789	1.000	0.867
300.5*	1.000	1.000–1.000	0.933	0.833–1.000	0.938	1.000	0.967
301.5	0.933	0.833–1.000	1.000	1.000–1.000	1.000	0.938	0.967
304	0.700	0.533–0.867	1.000	1.000–1.000	1.000	0.769	0.850
306.5	0.633	0.467–0.800	1.000	1.000–1.000	1.000	0.732	0.817
308	0.367	0.200–0.533	1.000	1.000–1.000	1.000	0.612	0.683
314.5	0.333	0.167–0.500	1.000	1.000–1.000	1.000	0.600	0.667
Inf	0.000	0.000–0.000	1.000	1.000–1.000	-	0.500	0.500

* Highest average of sensitivity and specificity. *AUC* = 0.998; 95% CI = 0.993–1.000.
